# Association of interleukin-6 with suicidal ideation in veterans: a longitudinal perspective

**DOI:** 10.3389/fpsyt.2023.1231031

**Published:** 2023-09-15

**Authors:** Shengnan Sun, Caroline M. Wilson, Sharon Alter, Yongchao Ge, Erin A. Hazlett, Marianne Goodman, Rachel Yehuda, Hanga Galfalvy, Fatemeh Haghighi

**Affiliations:** ^1^Nash Family Department of Neuroscience, Icahn School of Medicine at Mount Sinai, New York, NY, United States; ^2^James J. Peters VAMC, Bronx, NY, United States; ^3^Department of Neurology, Icahn School of Medicine at Mount Sinai, New York, NY, United States; ^4^Department of Psychiatry, Icahn School of Medicine at Mount Sinai, New York, NY, United States; ^5^Department of Psychiatry and Department of Biostatistics, Columbia University, New York, NY, United States

**Keywords:** veteran, suicidal ideation, suicidal behavior, depression, longitudinal, inflammation, IL-6, TNF-α

## Abstract

**Introduction:**

Studies showing associations between inflammation in suicide are typically cross-sectional. Present study investigated how cytokine levels track with suicidal ideation and severity longitudinally.

**Methods:**

Veterans with a diagnosis of major depressive disorder (MDD) with or without suicide attempt history (MDD/SA *n* = 38, MDD/NS *n* = 41) and non-psychiatric non-attempter controls (HC *n* = 33) were recruited, MDD/SA and HC groups were followed longitudinally at 3 months and 6 months. Blood plasma was collected and processed using Luminex Immunology Multiplex technology.

**Results:**

Significant differences in depression severity (BDI) and suicidal ideation severity (SSI) were observed across all groups at study entry, wherein MDD/SA group had the highest scores followed by MDD/NS and HC, respectively. Cytokines IL-1β, IL-4, TNF-α, IFN-γ, and IL-6 were examined at study entry and longitudinally, with IL6 levels differing significantly across the groups (*p* = 0.0123) at study entry. Significant differences in changes in cytokine levels between depressed attempters and the control group were detected for IL-6 (interaction *F*_1,91.77_ = 5.58, *p* = 0.0203) and TNF-α (*F*_1,101.73_ = 4.69, *p* = 0.0327). However, only depressed attempters showed a significant change, in IL-6 and TNF-α levels, decreasing over time [IL-6: *b* = −0.04, 95% CI = (−0.08, −0.01), *p* = 0.0245 and TNF-α: *b* = −0.02, 95% CI = (−0.04, −0.01), *p* = 0.0196]. Although IL-6 levels were not predictive of suicidal ideation presence [OR = 1.34, 95% CI = (0.77, 2.33), *p* = 0.3067], IL-6 levels were significantly associated with suicidal ideation severity (*b* = 0.19, *p* = 0.0422).

**Discussion:**

IL-6 was not associated with presence of suicidal ideation. IL-6 however, was associated with severity of ideation, suggesting that IL-6 may be useful in clinical practice, as an objective marker of heightened suicide risk.

## 1. Introduction

Globally, approximately 700,000 individuals died by suicide in 2021, and it is estimated that a suicide occurs every 40 s somewhere in the world (1). In the US, suicide is the 12th leading cause of death with the rate of death rising approximately 30% between 2010–2020 (2). The Veteran population suffers from even higher suicide rates. While comprising <10% of the population, veterans account for nearly 15% of deaths by suicide (3). Despite the VA’s implementation of extensive suicide prevention services nationwide, there were >6,000 veteran suicide deaths annually from 2008 to 2016, averaging 20 per day (4). Significant challenges remain in identifying individuals at high-risk for targeted suicide prevention and intervention (5), with current prognostic indicators that rely on psychosocial factors showing limited reliability (6, 7).

Emerging research has begun to investigate whether biological markers can contribute to improvement in suicide risk assessment, as well as contribute to greater understanding of the pathophysiology of suicidal ideation and behavior (8). A growing body of evidence shows associations between neuro and peripheral inflammation contributing to the pathophysiology of suicidality, including suicidal ideation, suicide attempt, and death by suicide (9–11). In a meta-analysis of 18 studies elevated levels of inflammatory mediators such as IL-6 and IL-1 β were consistently observed in blood and post- mortem brain samples of patients who died by suicide (9). Based on studies involving patients who receive interferon (IFN)-based or IL-2 immunotherapy (12–14), inflammation can trigger depressive symptoms and is associated with elevated suicide risk. A powerful retrospective study of 7.2 million Danes found that hospitalization for infection, and subsequent inflammation, increased the subsequent risk of suicide in a dose-dependent manner, by 42% overall (15). Data from a large perspective cohort study revealed that individuals with specifically higher CRP cytokine expression were 4.2 times more likely to die by suicide (16).

Majority of studies that show changes in cytokine levels are cross-sectional involving studies of brains from individuals who died by suicide, and of blood and cerebrospinal fluid (CSF) from living individuals who had previously attempted suicide (10, 15, 17–22). Across these and other studies converging evidence implicate cytokines interleukin-1 beta (IL-1β), interleukin-4 (IL-4), interleukin-6 (IL-6), interferon gamma (IFN-γ) and tumor necrosis factor-alpha (TNF-α) to be consistently associated with suicidal ideation and attempt history (11, 23, 24). However, longitudinal studies that investigate the dynamics of inflammation and cytokine expression in association with suicide risk and ideation are limited. In this longitudinal study, we investigated whether any of these suicide associated cytokines differed in their expression over time at recruitment entry (denoted as T0), 3 months, and 6 months timepoints alongside clinical measures of depression and suicide severity. Participants recruited at study entry included veterans with current depression with or without suicide attempt history (MDD/SA or MDD/NS), and veterans with no psychiatric condition or history of suicidal behavior (HC, considered as controls). Comparing these three groups at study entry, firstly we hypothesized that suicide attempt history confers a greater inflammatory milieu, even amongst veterans with current depression when comparing those with or without history of suicide attempt. Secondly, we hypothesized that amongst veterans with current depression and suicide attempt history changes in levels of these cytokines differ from controls longitudinally, and thirdly we hypothesized that these cytokine changes correlate with depression and suicide ideation severity overtime.

## 2. Method

All procedures contributing to this work comply with the ethical standards of the relevant national and institutional committees on human experimentation and with the Helsinki Declaration of 1975, as revised in 2008. All procedures involving human subjects/patients were approved by James J. Peters Veterans Affairs Medical Center (JJPVA) Institutional Review Board (IRB), approval number 1600366.

### 2.1. Study design and participant demographics

This longitudinal study was conducted with US veterans recruited from the JJPVA Medical Center in Bronx, New York. Participant recruitment was conducted through IRB-approved flyers, local advertisements, physician referrals, and community outreach. To determine potential candidates for inclusion in the study, participants were screened by research coordinators for eligibility screening. In order to be eligible for the study, participants had to have veteran status defined as having served for any length of time in any US military service branch, adult age (i.e., 18 to 80 years inclusive), proficiency with English language, and able to understand and complete informed consent procedures. Three groups were defined: (1) veterans with current depression and a diagnosis of major depressive disorder and history of suicide attempt (MDD/SA); (2) veterans with no history of psychiatric diagnosis or suicide attempt (HC), and a reference group (3) consisting of veterans with current depression and diagnosis of major depressive disorder and no history of suicide attempt (MDD/NS). A total of 211 interested participants were screened, out of which 112 individuals met criteria for enrollment. Participants with cancer, inflammatory illnesses, or who were taking pro-or-anti-inflammatory medications were screened out, to avoid confounds in cytokine results. Written consent was obtained by all enrolled participants and given to the clinicians prior to any clinical measures being administered. Due to funding constraints, only the HC and MDD/SA groups were followed longitudinally at 3 months and 6 months follow-up timepoints (*n* = 71).

### 2.2. Clinical measures

The Mini-International Neuropsychiatric Interview (M.I.N.I.) (25) was used to establish current major depressive disorder (MDD) diagnosis, meeting study inclusion. The M.I.N.I. is a structured diagnostic interview for DSM-IV disorders, designed to meet the need for a short but accurate structured psychiatric interview (25). The good psychometric properties of the MINI make it ideally suited for research purposes, and the brevity of the interview reduces participant burden (26). Participants who met criteria for serious mental illness (e.g., psychotic disorders or bipolar disorders) were excluded, as well as individuals with a history of traumatic brain injury (TBI) in the last 6 months to avoid confounds in inflammatory states related to injury. Suicide attempt status during the participant’s lifetime was determined using the Columbia Suicide History Form (CSHF) (27). The CSHF is a semi-structured instrument that characterizes lifetime suicide attempts, including method, lethality, precipitant, and surrounding circumstances of the event (28). In the present study, we qualify suicide attempt as any self-injurious behavior (including aborted and interrupted attempts) with the intent to end one’s life. Regardless of the lethality and method of the attempt, the intent to die was present. The CSHF was not used to obtain a score, but rather to ascertain individuals who have attempted suicide. These assessments were used to identify participants across the three groups (i.e., MDD/NS, MDD/SA, and HC).

All clinical interviews were conducted by trained Master’s level clinicians. Interrater reliability was established during weekly meetings under the supervision of MD-level clinical investigators. Additional validated self-report questionnaires were given to all participants to evaluate depression severity and current suicidal state. Depression severity was assessed by the Beck depression inventory version II (BDI), which is a 21-item self-report measure of characteristic attitudes and symptoms of depression (29). A meta-analysis of the BDI’s internal consistency yielded a Cronbach’s alpha of 0.86 for psychiatric patients and 0.81 for nonpsychiatric subjects (29). The Beck scale for suicidal ideation (SSI) was used to measure suicidal ideation. The SSI is a 21-item self-report questionnaire measuring thoughts of suicide (30). Participants rate each question using a Likert scale ranging from 0 to 2 (a rating of intensity for each respective item) based on how they felt during the most recent crisis and how they currently feel. Higher scores on the SSI indicate greater risk for suicide. The SSI has demonstrated good internal consistency among a sample of active duty service members (30) (*α* =0.89). Test–retest reliability over 1 week has demonstrated good reliability among a sample of inpatients (*α* = 0.88) (31). In addition, the SSI has demonstrated good predictive and discriminant validity (30). As part of the longitudinal study these assessments were administered at T0 for all three groups, and 3 months and 6 months for HC and MDD/SA groups which we followed longitudinally, with blood samples also collected at each timepoint for inflammatory assays.

### 2.3. Cytokine sample processing via Luminex

All blood draws were performed by trained phlebotomists. Blood samples were collected using EDTA tubes and processed to separate plasma for cytokine assays for each time point. Plasma was typically isolated within 2 h of blood collection and stored at −20°C prior to use. For inflammatory markers, we assayed cytokines, using the Milliplex Human Cytokine/Chemokine MAGNETIC BEAD Pemixed 41 Plex Kit (HCYTMAG-60K-PX41) that included IL-1β, IL-4, IL-6, IFN-γ and TNF-α, optimized for human serum, plasma and cell culture samples. Samples were assayed according to the manufacturer’s recommendations with modifications. Briefly, antibodies covalently attached to fluorescence-labeled magnetic beads were added to a 96-well plate. Samples of known concentrations of cytokine/chemokine standards (R&D Systems or Peprotech) were added to the wells containing the mixed antibody-linked beads and incubated at room temperature for 1 h followed by overnight incubation at 4°C with shaking at 500–600 rpm. Following the overnight incubation, plates were washed three times and then biotinylated detection antibodies were added at twice the recommended concentration for ELISA for 60 min at room temperature with shaking. Plates were washed as above and streptavidin-PE was added. After incubation for 30 min at room temperature, washes were performed as above, and reading buffer was added to the wells. Each sample was measured in duplicate. Plates were then read using a Luminex MAGPIX instrument with a lower bound of 50 beads per sample per cytokine. A total of 3,000 beads is used for each cytokine or chemokine per sample. The median fluorescence intensity (MFI) for each bead is determined for analysis with Milliplex software using a five-parameter regression algorithm. Median normalization was applied on the MFI for each cytokine to account for batch effect by scaling the cytokine MFI to the same median value across all batches.

### 2.4. Data & statistical analyses

Analyses were performed with the statistical language R version 4.0.3 (32). The MFI values for the five cytokines of interest, including IL-1β, IL-4, IL-6, IFN-γ and TNF-α were log transformed, and were graphed by group and time, and inspected for outliers and inconsistent values. Outliers, defined as values outside 1.5 times the interquartile range above the 3rd and below the 1st quartiles, were winsorized—censored to the nearest non-outlier value to avoid deleting several observations from the analysis (33).

Data missingness was assessed across the three timepoints and groups. Data missingness was considered in two categories: data missing at baseline due to non-report or missing assay, and data missing longitudinally due to missing a follow-up assessment or dropout. For the two groups with available blood specimens and cytokine data longitudinally, number of participants by timepoint, is as follows: MDD/SA (T0 *n* = 37, 3-month *n* = 24, 6 months *n* = 19) and HC (T0 *n* = 32, 3 months *n* = 28, 6-month *n* = 24). To determine whether missing data were associated with variables of interest, i.e., cytokine measures, BDI or SSI scores at study recruitment (T0), mixed effect models were fit separately, testing whether cytokine levels or BDI/SSI scores are predictive of whether the subject missed a subsequent assessment(s) at any time point, and found that for both groups, data missingness was not associated with T0 cytokine levels nor BDI or SSI scores. Thus, subsequent analyses were not weighted in any way to balance out missingness. It should be noted that mixed effect models can analyze data sets with data missing at random.

Participants’ demographic and clinical characteristics at T0 including age, sex, race, SSI score, BDI score, and the levels of the five cytokines of interest were reported in Table 1 and compared among the three groups using ANOVA followed by post-hoc Tukey HSD test for continuous measurements and Fisher’s exact for count measurements. For SSI, Wilcoxon rank sum test was used to compare MDD/SA to MDD/NS. Participants’ BMI was compared among the three groups using Kruskal Wallis test and within each group, and association between BMI and the five cytokine levels were assessed using Spearman correlation. No difference was observed in participant’s BMI across the three groups and no significant association between BMI and the five cytokines were observed in HC and MDD/SA groups. As such, BMI was not included in statistical models and analyses. Next, longitudinal analyses were performed for HC and MDD/SA groups. Linear mixed effect models with subject-specific random intercept were fit for the following analyses: first, we tested whether the association between cytokine levels, measured as log MFI, and time, defined as a continuous variable ranging from 0–6, differed by group, where separate models were fit for each of the five cytokines of interest, with time and group as main effect and their interaction in the model. Second, if significant time by group interaction was detected, we tested the association with time for the two groups separately. Otherwise, the interaction term was removed, and the association was tested using additive models. Third, when cytokine levels exhibited significant change over time (i.e., there was a significant change in slope for time per cytokine) in the MDD/SA group, associations between the specific cytokine and SSI and BDI, respectively, were assessed in the MDD/SA group (where SSI or BDI was the response and each cytokine’ the independent variable). Change in suicide ideation and depression severity longitudinally (i.e., SSI and BDI scores by time) were tested in the same manner as cytokine analyses above. For all models tested, age and sex were adjusted as a covariate in the model, except for models with HC group where only age was adjusted because of disproportionate sex distribution in the HC group. Type III ANOVA with Satterthwaite’s method (34) results were reported for the interaction models in Table 2. For all the models, either ANOVA or coefficient (*b*) with *p*-values and 95% CI from bootstrap (seed = 20210104) were reported. To address the high number of zero SSI observations, zero-inflated negative binomial mixed effect models were fit using glmmTMB package (35) with SSI scores as the response variable. Zero inflated models generate two sets of results, one for the zero-inflation part, and a second one for the severity, conditional on non-inflation. Coefficient (*b*) and *p*-value were reported for conditional model and odds ratio (OR) and *p*-value were reported for the zero-inflation model.

**Table 1:** Demographics and clinical information at recruitment entry including age, sex, race, BMI, and clinical measures for 112 participants in the present study.

	Total *N* = 112	MDD/SA *N* = 38	MDD/NS *N* = 41	HC *N* = 33	*p*-value	Sig. pair
**Age**, mean ± SD	49.2 ± 14.1	46.8 ± 12.3	51.3 ± 11.1	49.5 ± 18.7	0.3654	
**Sex**, *n* (%)						
Male	96 (86%)	31 (82%)	33 (80%)	32 (97%)	0.0679	
Female	16 (14%)	7 (18%)	8 (20%)	1 (3%)		
**Race**, *n* (%)						
White Non-Hispanic	24 (21%)	6 (16%)	6 (15%)	12 (36%)	0.1257	
Black Non-Hispanic	48 (43%)	15 (39%)	19 (46%)	14 (42%)		
Hispanic	32 (29%)	12 (32%)	13 (32%)	7 (21%)		
Other	8 (7%)	5 (13%)	3 (7%)	0 (0%)		
**BMI**, mean ± SD	29.9 ± 6.0	29.5 ± 5.0	30.5 ± 7.9	29.7 ± 5.1	0.9940^b^	
**Beck scale for suicide ideation (SSI)**, median (IQR)	0.0 (0.0–9.0)	13.0 (0.0–22.5)	0.0 (0.0–1.0)	0.0 (0.0–0.0)	<0.0001^b^	MDD/SA > MDD/NS, MDD/SA > HC, MDD/NS > HC
**Beck depression inventory (BDI)**, mean ± SD	20.2 ± 15.6	31.4 ± 12.5	23.7 ± 11.8	3.0 ± 3.7	<0.0001	MDD/SA > MDD/NS, MDD/SA > HC, MDD/NS > HC
**Cytokine level [log(MFI)]**^a^, mean ± SD						
IL-1β	3.7 ± 0.7	3.8 ± 0.8	3.8 ± 0.8	3.6 ± 0.6	0.3249	
IL-4	3.2 ± 0.7	3.3 ± 0.9	3.2 ± 0.7	3.1 ± 0.5	0.5505	
IL-6	4.1 ± 0.7	4.4 ± 1.0	3.9 ± 0.4	4.0 ± 0.6	0.0123	MDD/SA > MDD/NS
IFN-γ	3.3 ± 0.4	3.3 ± 0.4	3.2 ± 0.4	3.3 ± 0.4	0.5834	
TNF-α	4.3 ± 0.3	4.3 ± 0.3	4.3 ± 0.4	4.3 ± 0.4	0.7946	

a Cytokine data were available for 110 subjects; missing for 1 HC and 1 MDD/SA subjects.

b Kruskal–Wallis test followed by *post-hoc* pair-wise Wilcoxon rank sum test with Bonferroni correction. BDI scores were missing for 4 participants (1 MDD/SA, 2 MDD/NS and 1HC). SSI scores were missing for 10 participants (3 MDD/SA and 7 MDD/NS).

**Table 2:** Type III analysis of variance table with Satterthwaite’s method for the mixed effect models with random intercept for each subject and fixed effects of time, group and their interaction adjust for age on the four cytokines.

	IL-6		TNF-α		IL-4		IFN-γ		IL-1β	
	*F*	*p*	*F*	*p*	*F*	*p*	*F*	*p*	*F*	*p*
Age	0.00	0.9767	0.99	0.3243	0.88	0.3530	0.45	0.5057	0.09	0.7595
Timepoint	3.61	0.0605	1.30	0.2570	0.19	0.6676	0.19	0.6602	0.91	0.3433
Group	5.59	0.0207	0.38	0.5414	0.94	0.3363	0.18	0.6712	1.73	0.1906
Timepoint × group	5.58	0.0203	4.69	0.0327	0.31	0.5805	0.01	0.9219	0.94	0.3343

## 3. Results

### 3.1. Demographic and clinical comparison across three groups at recruitment entry (T0)

Subjects’ demographic and clinical assessment data are presented in Table 1. The three groups did not significantly differ on age (*p* = 0.3654). The study participants had an average age of 49.2 years (SD = 14.1). Overall, 16 (14%) of the participants were female. Although there was only 1 (3%) female in HC group, there was no statistically significant difference across the three groups by sex (Fisher’s exact test *p* = 0.0679). No statistically significant difference in race/ethnicity (*p* = 0.1257) was observed across the three groups. Depression severity as measured by BDI scores at T0 did significantly differ across the three groups (*p* < 0.0001; MDD/SA: mean = 31.4, SD = 12.5; MDD/NS: mean = 23.7, SD = 11.8; HC: mean = 3.0, SD = 3.7; Table 1), with depressed attempters showing significantly higher depression severity compared to both the depressed non-suicide (*p* = 0.0160) and HC (*p* < 0.0001) groups, and the depressed non-suicide group had significantly higher depression severity compared to the HC group (*p* < 0.0001); overall reflecting a stepwise pattern across the three groups. There was also a significant group difference for T0 SSI across the three groups (*p* < 0.0001; MDD/SA: median = 13.0, IQR =0.0–22.5; MDD/NS: median = 0.0, IQR = 0.0–1.0; HC: median = 0.0, IQR = 0.0–0.0; Table 1), MDD/SA group showed significantly higher ideation score compared to both MDD/NS group (*p* < 0.0001) and HC group (*p* < 0.0001), and MDD/NS group also showed significantly higher ideation score compared to HC group (*p* = 0.0023).

### 3.2. Comparison of cytokine levels across three groups at study entry

Cytokine measures for IL-1β, IL-4, TNF-α, and IFN-γ did not vary between the three groups at T0 (Table 1). However, IL-6 levels did significantly differ between the groups (*p* = 0.0123, Table 1). Post-hoc pairwise analysis showed that IL-6 levels were significantly higher in the MDD/SA group compared to the MDD/NS group (mean diff = 0.44, *p* = 0.0161); but no group differences were found between MDD/SA and HC (mean diff = 0.40, *p* = 0.0509) nor between MDD/NS and HC (mean diff = −0.05, *p* = 0.9586).

### 3.3. Longitudinal cytokine data analyses

**Figure 1:**
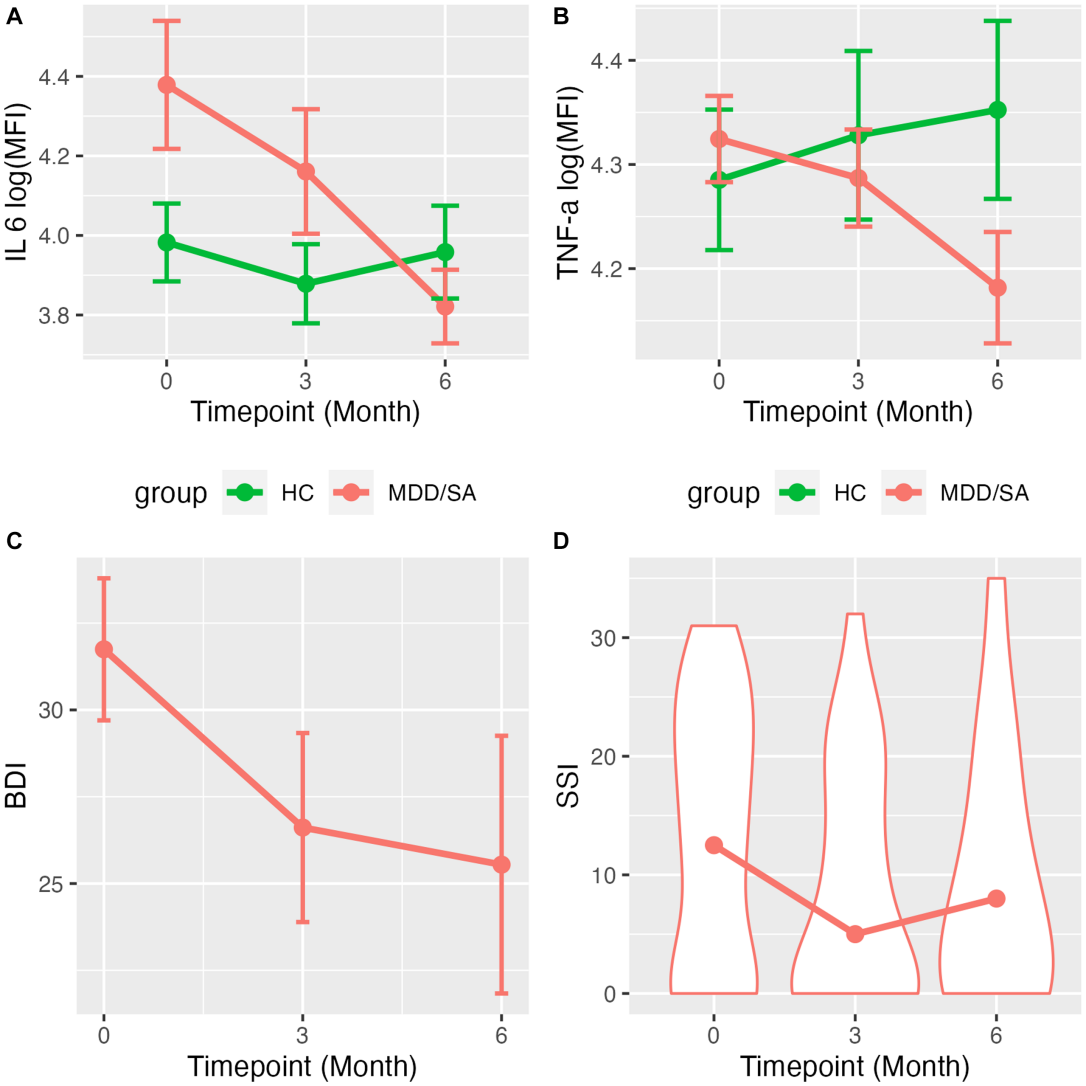
Line charts/violin plots show the average trajectory of cytokines levels/clinical measurements over time. Mean and standard error of log(MFI) across time for **(A)** IL-6 and **(B)** TNF-α by suicide group. **(C)** Mean and standard error of BDI scores across time for MDD/SA subjects. **(D)** Violine plots with medium of SSI score distribution across time for MDD/SA subjects.

We first examined whether the change in cytokine levels over time (timepoints: T0, 3 months, 6 months as a continuous variable) differed between the HC and MDD/SA groups using an interaction model. We found a significant Group × Time interaction for IL-6 (*F*_1,91.77_ = 5.58, *p* = 0.0203, Table 2 and Figure 1A) and TNF-α (*F*_1,101.73_ = 4.69, *p* = 0.0327, Table 2 and Figure 1B). Within the HC group, levels of IL-6 or TNF-α did not vary over time (IL-6: *F*_1,50.95_ = 0.19, *p* = 0.6636; TF-α: *F*_1,52.37_ = 0.47, *p* = 0.4951). However, in the MDD/SA group, levels of these cytokines significantly decreased over time [IL-6: *b* = − 0.04, 95% CI = (−0.08, −0.01), *p* = 0.0245; TNF-α: *b* = −0.02, 95% CI = (−0.04, −0.01), *p* = 0.0196]. It should be noted that no significant Group × Time interaction (Table 2) were detected for cytokines IL-1β (*F*_1,113.36_ = 0.94, *p* = 0.3343), IL-4 (*F*_1,91.29_ = 0.31, *p* = 0.5805) or IFN-γ (*F*_1,97.58_ = 0.01, *p* = 0.9219). When changes in levels of IL-1β, IL-4 or IFN-γ were assessed longitudinally using additive models without interaction, no significant changes over time were detected (IL-1β: *F*_1,113.36_ = 1.04, *p* = 0.3103, IL-4: *F*_1,92.11_ = 0.14, *p* = 0.7096, IFN-γ: *F*_1,97.98_ = 0.19, *p* = 0.6509).

### 3.4. Longitudinal association between disease severity and cytokine data

Depression severity showed a significant decline over time in the MDD/SA group [BDI: *b* = −1.01, 95% CI = (−1.93, −0.09), *p* = 0.0376], as shown in Figure 1C. Although there was no significant change in the proportion of MDD/SA participants who ideated over time in the zero-inflation part of the model (OR = 1.02, 95% CI: 0.83–1.25, *p* = 0.8554), conditional on ideation, suicide ideation severity decreased significantly over time (*b* = −0.06, *p* = 0.0100), as shown in Figure 1D. Further, within the MDD/SA group, we investigated the association between IL-6 and TNF-α on one hand (as a predictor) and BDI and SSI on the other (as a response), and found no significant associations longitudinally between BDI scores and the two cytokines [BDI and IL-6: *b* = 3.25, 95% CI = (−0.89, 7.26), *p* = 0.1234, or BDI and TNF-α: *b* = 8.07, 95% CI = (−5.07, 20.94), *p* = 0.2033]. IL-6 expression was not predictive of suicidal ideation presence (OR = 1.34, 95% CI = 0.77–2.33, *p* = 0.3067). However, IL-6 expression was associated with ideation severity, wherein decrease in IL-6 levels conditioned on suicide ideation presence was significantly associated with the reduction in severity of ideation (SSI) (*b* = 0.19, *p* = 0.0422). Lastly, SSI scores were not associated with TNF-α levels longitudinally (conditional model *b* = 0.56, *p* = 0.0708; zero-inflation model OR = 2.76, 95% CI = 0.40–18.98, *p* = 0.3022).

## 4. Discussion

Given emerging data linking inflammation is a potential risk factor for suicidal thoughts and behavior, the primary aim of this study was to investigate the association between IL-1β, IL-4, IL-6, IFN-γ and TNF-α cytokines that have been previously implicated in suicidality using a longitudinal design in a veteran cohort. Comparison of cytokine levels between MDD attempters and controls longitudinally showed significant Time by Cytokine associations for both IL-6 and TNF-α. However, only IL-6 levels showed a positive association with suicidal ideation severity longitudinally, specifically amongst those veterans at risk with current depression and history of suicide attempt with elevated suicidal ideation at study entry. Interestingly, at study entry, when comparing veterans with MDD diagnosis with suicide attempt history (MDD/SA) vs. those with MDD and no suicide attempt (MDD/NS), significant difference in IL-6 levels were detected, with MDD attempters having elevated levels of IL-6 protein expression. This underscores the significance of the IL6 findings to be suicide specific, and not likely driven by MDD psychopathology.

Limitations to our study include the small sample size, especially in the longitudinal time points due to attrition, as well as lack of follow up data across in the MDD/NS group. Also, the large variance seen in the cytokine measurements is another limitation. Further, the timing of the blood draws was not standardized, i.e., not drawn in a fasting state and at the same time of the day for every participant longitudinally. This may have confounded our IL-6 findings, since IL-6 secretion follows a circadian rhythm (36). Participants were excluded in this study with cancer, inflammatory illnesses, or who were taking pro-or-anti-inflammatory medications that would have confounded cytokine results. However, no other health factors were exclusionary, and as such were not assessed, given the transdiagnostic nature of suicide that cuts across diagnoses, while still keeping the study findings generalizable to the at-risk veteran populations with complex comorbid medical and mental health conditions. There were also many strengths to the study design including the longitudinal nature of the study, use of well-validated clinical assessments, immunological parameters were evaluated across all participants, and in-depth phenotyping of suicidal ideation and behavior.

Overall, results from this study are consistent with prior observations of high IL-6 associations with suicidal ideation and history of attempt. Immune system dysregulation has already been associated with suicidal symptomatology, with increases in expression of proinflammatory cytokines (9, 11, 37–41). The most consistent finding across these and other studies, is the elevation of IL-6 cytokine levels in patients with suicidal ideation or behavior, as compared to those with no suicidality or HCs. Furthermore, in postmortem human brain studies, significant increase in levels of IL-6 mRNA and protein expression have been detected in prefrontal cortex of suicide decedents as compared to controls (22, 42). Greater severity in suicidal symptoms have been associated with elevated CSF IL-6 levels in patients with history of suicide attempt (43). Elevated IL-6 levels in blood have similarly been reported in suicide attempters, compared with non-suicidal depressed patients, and HCs (17), with blood IL-6 levels distinguishing psychiatric patients with suicidality, compared to psychiatric patients without suicidality and HCs (9). Taken together, these data point to a unique immuno-biological signature, with elevated IL-6 levels being linked to increased suicide risk (23).

As a proinflammatory cytokine involved in induction of molecular cascades and processes of acute inflammation, IL-6 also plays an important role in the physiological homeostasis of the brain with elevated IL-6 expression affecting neuronal plasticity, neurogenesis, and neurotransmission (44–47). IL-6 has been implicated in the pathogenesis of neurodegenerative disorders with profound neuropathological changes; including Alzheimer’s, Parkinson’s, and multiple sclerosis; where elevated IL-6 expression have been observed (48). IL-6 has also been linked to a number of psychiatric disorders such as anxiety, depression, and of relevance to the present study, suicidal ideation and behavior (49). As such, inflammation is a promising candidate for interventional modification, since changes in cytokine expression and specifically, activation of the proinflammatory IL-6 cytokine can confer some influence over suicidal risk and behaviors in predisposed individuals [reviewed in Ganança et al. (11)].

To conclude, in recent years, a number of studies have identified biological markers associated with suicide risk, however longitudinal studies are essential to establish the complex relationship between inflammation and suicide risk severity. Identification of potential biomarkers that are modifiable and track with clinical outcomes has significant clinical importance for patients at high risk for suicide. In the current naturalistic study, among the veterans at highest risk with current depression and history of suicide attempt, decreases in levels of suicidal ideation were significantly associated with decreases in IL-6 levels over 6 months of observation. This suggest that targeting the inflammatory system may provide a novel therapeutic approach for the pharmacotherapy of depression and suicide prevention, with the goal of improved detection and treatment of patients at elevated risk for suicide. These findings have important clinical implications, underscoring the role of IL-6 as an accessible peripheral candidate biomarker with potentially predictive value for determination of the severity of suicide risk.

## Data availability statement

The original contributions presented in the study are included in the article/supplementary material, further inquiries can be directed to the corresponding author.

## Ethics statement

The studies involving humans were approved by James J. Peters Veterans Affairs Medical Center (JJP VAMC) Institutional Review Board (IRB), approval number 1600366. The studies were conducted in accordance with the local legislation and institutional requirements. The participants provided their written informed consent to participate in this study.

## Author contributions

FH, HG, YG, MG, EH, and RY contributed to conceptualization of study protocol. SA, MG, EH, and FH contributed to study recruitment, to phenotypic, and biospecimen collection. FH, HG, YG, CW, and SS contributed to study-design and conducted experiments. CW conducted sample processing and quality control. SS performed inflammation and symptom data analyses with statistical/analytical guidance from HG, and data normalization guidance from YG. SS, HG, and FH prepared the manuscript. All authors contributed to the article and approved the submitted version.

## Funding

FH is a recipient of the VA CSR&D Research Career Scientist Award; CX002074, and her laboratory and work is supported by CX001728, BX003794, and RX003818 at the James J. Peters VA Medical Center. EH is supported by a VA CSR&D Research Career Scientist Award (CX001738), a VA Merit (I01 CX001451), and Collaborative VA Merit (CX002093). MG is supported by the VISN 2 MIRECC, Collaborative VA Merit (CX002093), VA Merit (CX001705), VA SPiRE (RX004092), and a CSRD Suicide Prevention Resource Center grant.

## Conflict of interest

The authors declare that the research was conducted in the absence of any commercial or financial relationships that could be construed as a potential conflict of interest.

## Publisher’s note

All claims expressed in this article are solely those of the authors and do not necessarily represent those of their affiliated organizations, or those of the publisher, the editors and the reviewers. Any product that may be evaluated in this article, or claim that may be made by its manufacturer, is not guaranteed or endorsed by the publisher.
